# Hematopoiesis in the spleen after engraftment in unrelated cord blood transplantation evaluated by ^18^F-FLT PET imaging

**DOI:** 10.1007/s12185-023-03658-z

**Published:** 2023-10-02

**Authors:** Hiroaki Araie, Naoko Hosono, Tetsuya Tsujikawa, Yasushi Kiyono, Hidehiko Okazawa, Takahiro Yamauchi

**Affiliations:** 1 Department of Hematology and Oncology, Faculty of Medical Sciences, University of Fukui, 23-3 Matsuoka Shimoaizuki, Eiheiji-cho, Yoshida-gun, Fukui, 910-1193 Japan; 2 Department of Radiology, Faculty of Medical Sciences, University of Fukui, Fukui, Japan; 3 Biomedical Imaging Research Center, Faculty of Medical Sciences, University of Fukui, Fukui, Japan

**Keywords:** Allogeneic hematopoietic stem cell transplantation, ^18^F-FLT PET imaging, Cord blood transplantation, Hematopoiesis

## Abstract

Cord blood is an important donor source for allogeneic hematopoietic stem cell transplantation (allo-HSCT), with its unique composition and quality of hematopoietic cells. The proliferation site and potency of infused hematopoietic stem cells in humans may vary between stem cell sources. We investigated this possibility in a prospective, exploratory study to assess hematopoietic dynamics using the radiopharmaceutical 3′-deoxy-3′-^18^F-fluorothymidine (^18^F-FLT), a thymidine analog used in positron emission tomography imaging, before allo-HSCT and on days 50 and 180 after allo-HSCT. We evaluated 11 patients with hematological malignancies who underwent allo-HSCT [five with peripheral blood stem cell transplantation (PBSCT) and six with unrelated cord blood transplantation (UCBT)]. Before allo-HSCT, ^18^F-FLT uptake did not differ between the two groups. At day 50, ^18^F-FLT uptake in the spleen was significantly greater in the UCBT group than in the PBSCT group (*p* = 0.0043), with no difference in whole-body bone marrow. At day 180, the differences in spleen uptake had diminished, and there were no differences between groups in whole-body bone marrow or the spleen, except for the sternum. The persistence of splenic hematopoiesis after engraftment in the UCBT group may reflect the complex systemic homing and proliferation mechanisms of cord blood hematopoietic cells.

## Introduction

Unrelated cord blood transplantation (UCBT) is a valuable alternative donor source when a suitable related donor is unavailable for allogeneic hematopoietic stem cell transplantation (allo-HSCT) [1]. However, it is necessary to recognize the distinctive clinical course differences between UCBT and bone marrow transplantation (BMT) or peripheral blood stem cell transplantation (PBSCT) [2]. Specifically, UCBT is prone to delayed or failed engraftment and has an increased risk of non-relapse mortality related to infection [3, 4]. Furthermore, cellular immunodeficiency may persist after engraftment with increased frequency of viral infections [5, 6]. Conversely, chronic graft-versus-host disease (GVHD) is less frequent than in BMT/PBSCT [2], and the high graft-versus-leukemia effect of UCBT may reduce the risk of relapse [7, 8]. These factors are important in determining the success of transplantation.

The composition, quantity, and characteristics of cord blood cells differ from those observed in peripheral blood or bone marrow [9–11]. Cord blood cells exhibit an enhanced proliferative response to cytokines and a reduced dependence on stromal cells compared with their bone marrow or peripheral blood counterparts [12–14]. The modest degree of natural cytotoxicity observed in cord blood cells is attributed to the naive quality of the immune cells in cord blood [15–17]. These differences between cord blood and bone marrow or peripheral blood potentially affect the clinical course.

During BMT/PBSCT, transplanted hematopoietic stem cells (HSCs) participate in extramedullary hematopoiesis in the hepatic and splenic regions during the initial phase of transplantation, before the onset of hematopoiesis in the bone marrow [18–21]. However, it remains uncertain whether the site and intensity of HSC proliferation after allo-HSCT are similar for BMT/PBSCT and UCBT, where the quality of HSCs is different. Furthermore, the iliac crest bone marrow examination performed in routine practice cannot extrapolate whole-body hematopoiesis.

The radiopharmaceutical 3′-deoxy-3′-^18^F-fluorothymidine (^18^F-FLT), a thymidine analog used in positron emission tomography (PET) imaging, is a surrogate indicator of deoxyribonucleic acid synthesis [22]. ^18^F-FLT PET is a valuable method for assessing hematopoietic proliferative activity in the bone marrow and extramedullary space [20, 23, 24]. We have previously delineated the efficacy of combining ^18^F-FLT PET with magnetic resonance imaging (MRI) for the assessment of bone marrow failure and hematopoietic recovery following chemotherapy [25–27]. In this study, we aimed to evaluate the hematopoietic status in allogeneic transplant recipients using ^18^F-FLT PET/MRI, focusing on the differences between UCBT and BMT/PBSCT.

## Methods

### Study design and participants

We conducted a planned, prospective, open-label, exploratory study in a sub-cohort of patients enrolled in a study at the University of Fukui Hospital, “The impact of the source of stem cells on the kinetics of hematopoiesis and the outcome of allogeneic hematopoietic cell transplantation, evaluated using ^18^F-FLT PET/MRI” (UMIN000041491). Patients aged ≥ 20 years scheduled for allo-HSCT for hematologic malignancies (leukemia, myelodysplastic syndrome, myeloproliferative neoplasms, malignant lymphomas, and multiple myeloma) were included. This study was conducted following the guiding principles of the Declaration of Helsinki and approved by the Research Ethics Committee of Fukui University (number 20200041). Furthermore, written informed consent was obtained from all patients before enrollment. The primary endpoint of the study was the donor-specific assessment of pre- and post-transplant hematopoietic activity in the bone marrow and extramedullary space.

### Procedures

Patients’ hematopoietic status was assessed using ^18^F-FLT PET/MRI before allo-HSCT (from day 21 to the start of conditioning, with day 0 designated as the transplant date), at day 50 ± 14, and at day 180 ± 14. ^18^F-FLT preparation was performed according to previous reports [25–27]. Patients were treated with an intravenous injection of 185 MBq ^18^F-FLT. Fifty minutes after injection, patients were transported to a simultaneous whole-body 3.0 T PET/MR scanner (Signa PET/MR; GE Healthcare, Waukesha, WI), which provided anatomic coverage from the vertex to the mid-thigh. PET data were acquired in 3D mode at a 5.5 min/bed rate (89 slices/bed) in five–six beds with 24-slice overlap. The 5.5 min/bed rate was selected to accommodate the MRI sequences collected at each bed. At each bed position, a 2-point Dixon 3D volumetric interpolated T1-weighted fast spoiled gradient echo sequence (TR/TE1/TE2, 4.0/1.1/2.2 ms; field of view, 50 × 37.5 cm; matrix, 256 × 128; slice thickness/overlap, 5.2/2.6 mm; 120 images/slab; acquisition time, 18 s) was acquired and used to generate MR attenuation correction maps. The PET data were reconstructed using ordered subset expectation maximization by selecting 14 subsets and 3 iterations, and post-smoothed with a 3 mm Gaussian filter. The reconstructed images were converted into semi-quantitative images corrected for injection dose and participant’s weight (= standardized uptake value: SUV). To verify the relationship between FLT PET findings and clinical outcome, blood counts, lymphocyte fractions, immunoglobulin measurements, and bone marrow examinations were performed on days 50, 100, and 180.

### Statistical analysis

The following sites were analyzed on ^18^F-FLT PET/MRI images: Sternum (manubrium and sternal body), thoracic spine (Th 4‒6), lumbar spine (L 2‒4), bilateral iliac crest, long bones of the extremities (bilateral femoral shafts), spleen, and liver. For quantitative assessment, spherical regions of interest (ROIs) were applied in consultation with an experienced radiologist and hematologist. ROIs of 1 cm diameter were placed on the sternum, thoracic, lumbar, iliac, and femoral shaft at each of the above sites, mean SUVs were measured, and the mean values were used for analysis. ROIs of 3 cm and 5 cm in diameter were placed in the spleen and liver, respectively, and the mean SUV was used for analysis. Patients who did not have neutrophil engraftment after allo-HSCT were excluded from the study. Patients whose refusal to continue the study, relapse of the primary disease after transplantation, deterioration of their general health, or death prevented them from undergoing imaging assessments were censored at the appropriate time point. Patient characteristics, laboratory results, and ^18^F-FLT PET SUVs were compared using Fisher’s exact test for categorical variables, Pearson’s cumulative correlation coefficient, and Mann‒Whitney U test for continuous variables. Two-sided *p* values of < 0.05 were considered as statistically significant. All statistical analyses and graphs were performed using EZR version 1.55 (Saitama Medical Center, Jichi Medical University, Saitama, Japan), a graphical user interface for R (The R Foundation for Statistical Computing, Vienna, Austria, version 4.1.2) [28], and GraphPad Prism (version 9.3.1 for Windows, GraphPad Software, La Jolla California USA, www.graphpad.com).

## Results

### Patient background and transplantation outcome

From August 2020 to March 2023, 13 patients scheduled for allo-HSCT were enrolled. Two patients were excluded because of engraftment failure due to early relapse of the primary disease. Thus, 11 patients were analyzed and their characteristics are presented in Table 1. Five patients underwent PBSCT, six patients underwent UCBT, and no patient underwent BMT. Within the PBSCT cohort, two patients underwent haploidentical transplantation. One patient with malignant lymphoma in the PBSCT group was non-remission. The median number of CD34-positive cells infused was 4.8 × 10^6^ cells per kilogram of patient weight in the PBSCT group and 1.1 × 10^5^ cells per kilogram of patient weight in the UCBT group. The median engraftment time after transplantation was 13 days (range 10–23) in the PBSCT cohort and 21 days (range 15–39) in the UCBT cohort. Each patient underwent ^18^F-FLT PET/MRI on day 50 ± 14; however, two patients in the PBSCT group and one patient in the UCBT group could not undergo the procedure on day 180 ± 14 owing to relapse and deterioration in health status associated with acute GVHD. Four of six patients in the UCBT group had bacteremia prior to engraftment, but all had no complications of bacterial or cytomegalovirus infection at the time of ^18^F-FLT PET/MRI on day 50. There were no complicated cases of pre-engraftment immune reaction (PIR). During ^18^F-FLT PET/MRI on day 50, each patient was treated with tacrolimus for GVHD prophylaxis. In addition, two patients in the PBSCT group and one patient in the UCBT group were treated with systemic corticosteroid therapy for acute GVHD. During ^18^F-FLT PET/MRI on day 180, two patients in the PBSCT group were treated with tacrolimus.

**Table 1:** Patient characteristics

	PBSCT	CBT	*p* value
No. of patients, *N* (%)	5 (100)	6 (100)	
Age, median [range]	37 [23–60]	46 [27–65]	0.52
Sex (%)			
Female	2 (40.0)	3 (50.0)	1
Male	3 (60.0)	3 (50.0)	
Disease			
Myelodysplastic syndrome/acute myeloid leukemia	2 (40.0)	5 (83.3)	0.39
Acute lymphoblastic leukemia	1 (20.0)	1 (16.7)	
Malignant lymphoma	2 (40.0)	0 (0.0)	
Disease status			
Complete remission	4 (80.0)	6 (100.0)	0.45
Non-remission	1 (20.0)	0 (0.0)	
Conditioning			
Myeloablative	1 (20.0)	5 (83.3)	0.08
Reduced intensity	4 (80.0)	1 (16.7)	
Graft-versus-host disease prophylaxis			
Tacrolimus + short-term methotrexate	3 (60.0)	5 (83.3)	0.54
Tacrolimus + mycophenolate mofetil	0 (0.0)	1 (16.7)	
Tacrolimus + mycophenolate mofetil + post-transplantation cyclophosphamide	1 (20.0)	0 (0.0)	
Cyclosporine + mini-dose methotrexate + alemtuzumab	1 (20.0)	0 (0.0)	
CD34 stem cell dose (× 10^5^cells per kilogram)	28.8 [12.0, 84.0]	1.1 [0.9, 1.8]	0.006

PBSCT, peripheral blood stem cell transplantation; UCBT, unrelated cord blood transplantation

### Comparison of PBSCT and CBT on ^18^F-FLT PET/MRI and laboratory findings

**Fig. 1:**
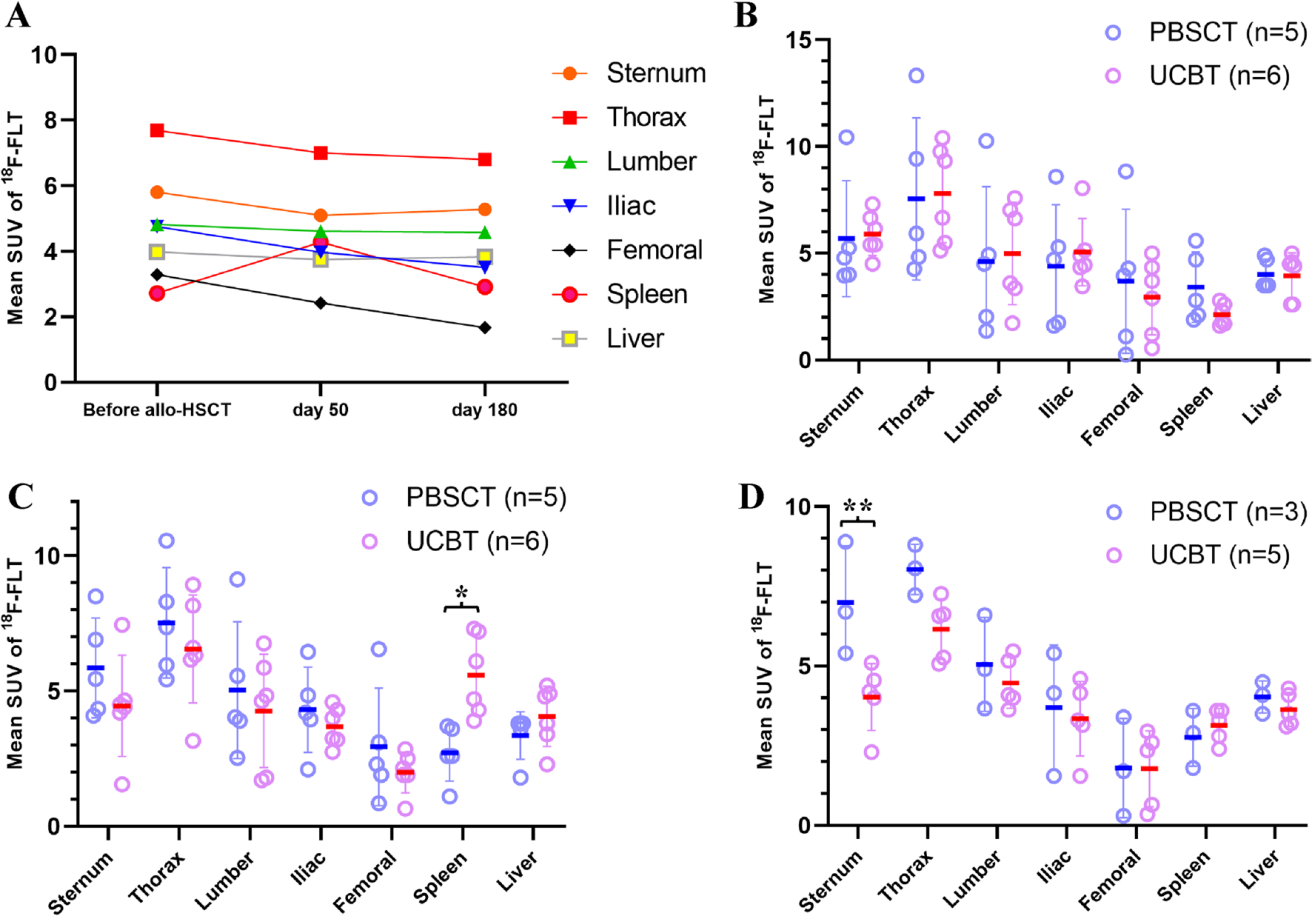
^18^F-FLT uptake in whole-body bone marrow and extramedullary hematopoiesis. **A** Mean SUV of ^18^F-FLT uptake of whole-body bone marrow and extramedullary hematopoiesis in all ten patients before allo-HSCT, on days 50 ± 14 and 180 ± 14. The uptake in the sternum, thoracic spine, lumbar spine, iliac crest, femoral shaft, and liver tended to level off to slightly decrease from pre- to post-transplant; however, only the uptake in the spleen increased at day 50 ± 14 and decreased at day 180 ± 14. **B**–**D** Comparison of mean SUV and standard deviation of ^18^F-FLT uptake between PBSCT and UCBT. The uptake was not significantly different in the sternum, thoracic spine, lumbar spine, iliac crest, femoral shaft, and liver before allo-HSCT (**B**), on day 50 ± 14 **(C**) and day 180 ± 14 (**D**). Spleen uptake was significantly higher during UCBT than PBSCT only on day 50 ± 14 (*p = 0.004); however, it was not different before allo-HSCT and on day 180 ± 14. Sternum uptake on day 180 ± 14 was significantly higher during PBSCT than UCBT (**p = 0.035). *Abbreviations* ^18^F-FLT, 3′-deoxy-3′-18F-fluorothymidine; allo-HSCT, allogeneic hematopoietic stem cell transplantation; PBSCT, peripheral blood stem cell transplantation; SUV, standardized uptake value; UCBT, unrelated cord blood transplantation

The median time between the last cytotoxic therapy and ^18^F-FLT PET/MRI before allo-HSCT was 28 days (range 17‒44) in the PBSCT group and 29.5 days (range 14‒76) in the UCBT group. The mean SUV of ^18^F-FLT uptake in all eleven patients in both groups was comparatively elevated in the thoracic spine (mean: 7.6 ± standard deviation: 2.9), followed by the sternum (5. 8 ± 1.8), lumbar spine (4.8 ± 2.8), iliac crest (4.7 ± 2.1), and femoral shaft (3.2 ± 2.5), with minimal uptake observed in the spleen (2.7 ± 1.2) (Fig. 1A). No significant differences in ^18^F-FLT uptake were observed between the PBSCT and UCBT groups at any site (Fig. 1B). There was no significant difference in spleen size before allo-HSCT between the PBSCT (median: 92.5 mm, range 70–96) and UCBT (median: 70.5 mm, range 57–85) groups (*p* = 0.051).

**Fig. 2:**
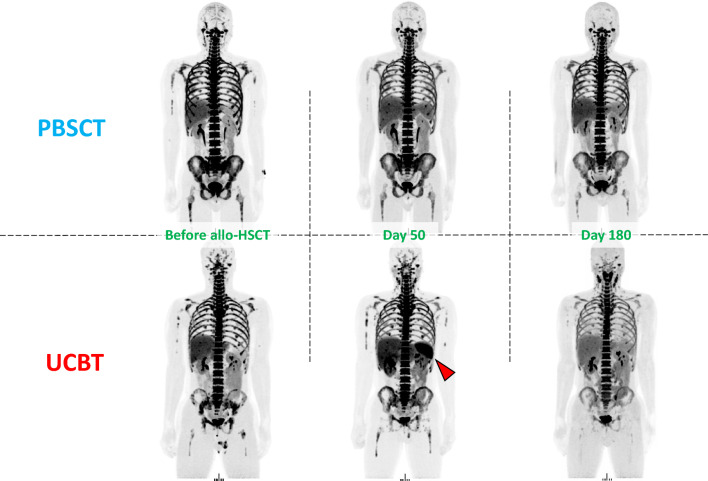
Representative maximum intensity projection ^18^F-FLT images of PBSCT and UCBT patients before allo-HSCT, on days 50 ± 14 and 180 ± 14. The PBSCT patient was a 29-year-old man with acute lymphoblastic leukemia in first complete remission. The UCBT patient was a 27-year-old man with acute myeloid leukemia in first complete remission. The arrows indicate the increased uptake in the spleen of the UCBT patient on day 50. *Abbreviations* ^18^F-FLT, 3′-deoxy-3′-18F-fluorothymidine; allo-HSCT, allogeneic hematopoietic stem cell transplantation; PBSCT, peripheral blood stem cell transplantation; SUV, standardized uptake value; UCBT, unrelated cord blood transplantation

At day 50 ± 14 ^18^F-FLT PET/MRI imaging was performed at a similar interval in both groups, with no significant difference between the PBSCT (median: 45 days, range 42–51) and UCBT (median: 48.5 days, range 42–58) groups (*p* = 0.64). The mean SUV of ^18^F-FLT uptake in the spleen showed a significant increase in the UCBT group compared with the PBSCT group (5.5 ± 1.4 vs. 2.7 ± 1.0, *p* = 0.0043). Conversely, the mean SUV of ^18^F-FLT uptake in the whole-body bone marrow increased in the PBSCT group compared with the UCBT group; however, the difference was not statistically significant (Fig. 1C). In the PBSCT group, the mean SUV was most prominent in the thoracic spine, followed by the sternum, lumbar spine, iliac crest, femoral shaft, and the spleen. Conversely, in the UCBT group, the mean SUV was most prominent in the thoracic spine, followed by the spleen, sternum, lumbar spine, iliac crest, and the femoral shaft (Fig. 1B). Visual assessment using maximum intensity projection images of ^18^F-FLT PET showed enhanced ^18^F-FLT uptake of the spleen in the UCBT group compared with the PBSCT group (Fig. 2). There was no significant correlation between the size of the spleen before allo-HSCT and ^18^F-FLT uptake in the spleen (correlation coefficient − 0.41, *p* = 0.20).

^18^F-FLT PET/MRI imaging was performed on day 180 ± 14 at a similar interval in both groups, with no significant difference between the PBSCT (median: day 185, range 175–189) and UCBT groups (median: day 182, range 171–189) (*p* = 0.36). There were no significant differences in ^18^F-FLT uptake between the PBSCT and UCBT groups at any site except the sternum, and the mean SUV of ^18^F-FLT uptake in the spleen for each group was decreased compared with that of whole-body bone marrow, excluding the femoral shaft (Fig. 1D).

The ^18^F-FLT SUVs of the liver, an organ capable of extramedullary hematopoiesis, were consistent before allo-HSCT, at days 50 and 180 (Fig. 1A), and there were no significant differences between the two groups at these time points (Fig. 1B–D).

Neutrophil, platelet, and reticulocyte counts, CD4 and CD8 lymphocyte counts, IgG levels, and bone marrow nuclear cell count did not change significantly between the two groups at days 50, 100, and 180 (Table 2). Eosinophil and CD20-positive B-lymphocyte counts did not change significantly between the two groups at day 50. However, the median eosinophil count was higher in the UCBT group than in the PBSCT group at day 100 [596 (range 0‒1200) vs. 63 (range 0‒111), *p* = 0.069] and was significantly higher in the UCBT group than in the PBSCT group at day 180 [598 (range 345‒1206) vs. 213 (range 79‒227), *p* = 0.025]. B-lymphocyte count was significantly higher in the UCBT group than in the PBSCT group at day 100 [897 (range 493‒1128) vs. 132 (range 9‒330), *p* = 0.014], and at day 180 [1005 (range 745‒1365) vs. 94 (range 93‒330), *p* = 0.025].

**Table 2:** Laboratory findings at day 50, day 100, and day 180 ± 14 after PBSCT or UCBT

	Day50			Day100			Day180		
	PBSCT (*n* = 5)	UCBT (*n* = 6)	*p* value	PBSCT (*n* = 4)	UCBT (*n* = 6)	*p* value	PBSCT (*n* = 3)	UCBT (*n* = 5)	*p* value
White blood cell (/µL)	7000 [3100–17800]	4850 [2300–10100]	0.36	5100 [2700–9000]	7550 [5000–8100]	0.39	5200 [3500–6100]	5700 [5000–6700]	0.45
Neutrophil (/µL)	6265 [1797.4–16020]	2528 [1058–7777]	0.36	3153 [1161–6930]	4165 [2530–4762.5]	0.52	2642 [1924–3702]	2453 [1372–2793]	0.29
Eosinophil (/µL)	83 [0–248]	89.5 [0–1470]	0.7	63 [0–111]	596 [0–1200]	0.069	213 [79–227]	598 [345–1206]	0.025
Lymphocyte (/µL)	527 [175–1166]	559 [101–881]	0.28	971 [585–1550]	1534 [850–2310]	0.088	1909 [332–1924]	2144 [1482–2772]	0.29
CD4-positive cell (/µL)	210 [146–278]	243 [116–742]	0.58	259 [105–330]	220 [117–368]	1	199 [169–330]	402 [214–739]	0.1
CD8-positive cell (/µL)	305 [23–423]	95 [51–256]	0.22	411 [190–731]	312 [126–340]	0.46	731 [259–1317]	338 [156–603]	0.29
B (CD20-positive) cell (/µL)	39 [5–205]	10 [0–486]	0.8	132 [9–330]	897 [493–1128]	0.014	94 [93–330]	1005 [745–1365]	0.025
Reticulocyte (× 10^4^/µL)	12.9 [3–17]	8 [0.8–10.8]	0.1	7.6 [6.7–20.8]	8.9 [3.9–14.8]	1	8.0 [5.4–10.7]	5.1 [3.0–8.3]	0.35
Platelet (× 10^4^/µL)	9.8 [2.4–16.6]	6.5 [4.4–20.5]	0.58	12.1 [9.8–17.7]	15.9 [10.4–18.1]	0.52	14.0 [12.1–16.5]	17.6 [5.2–21.1]	0.29
IgG (mg/dL)	693 [490–1059]	734 [145–858]	0.91	702 [319–1046]	698 [368–997]	1	626 [297–929]	811 [786–899]	0.7
Nuclear cell count In iliac crest (× 10^4^/µL)	5.4 [4.1–8.9]	4.8 [0.7–12]	0.8	5.4 [4.1–11.8]	4.9 [1.05–10.3]	0.52	5 [2.7–14]	3.3 [2.0–5.6]	0.65

PBSCT, peripheral blood stem cell transplantation; UCBT, unrelated cord blood transplantation

## Discussion

On day 50 after allo-HSCT, the UCBT group showed high ^18^F-FLT uptake in the spleen, with a mean SUV comparable to that of the thoracic spine, where uptake was strongest throughout the body. The increased uptake in the spleen was not observed in the PBSCT group at day 50, and the increased uptake in the spleen disappeared by day 180 in the UCBT group.

Hematopoiesis occurs in the spleen during the process leading to engraftment in mouse models of hematopoietic stem cell transplantation [18, 19, 21, 29]. In human BMT/PBSCT, ^18^F-FLT uptake in the spleen increases in the early post-transplant period (day 5) and decreases subsequently [20]. After engraftment, ^18^F-FLT uptake occurs predominantly in the sternum, thoracolumbar spine, and pelvic region, returning to the normal adult human hematopoietic state [20]. This pathway of human HSCs engraftment mirrors that of human fetal ontogeny; however, it is unclear whether it is similar in UCBT. To our knowledge, this study is the first to show that ^18^F-FLT uptake in the spleen is enhanced in UCBT even at approximately day 50 post-transplant, the period of immune reconstitution.

The spleen generates extramedullary hematopoiesis [30–32], and ^18^F-FLT PET detects extramedullary hematopoietic activity in the spleen [24, 25]. The bone marrow stromal niche that supports hematopoiesis is also present in the spleen, but its structure differs in that there are no osteoblasts in the spleen [33–35]. High-intensity chemotherapy and irradiation administered as conditioning for allo-HSCT can damage not only the patient’s own hematopoietic cells, but also the stromal cells that make up the bone marrow niche [36]. Restoration of the bone marrow niche is essential for reconstitution of hematopoiesis by donor-derived hematopoietic stem cells. The spleen niche is also thought to be compromised by conditioning, but the spleen continuously remodels its stromal microenvironment to adapt to the immune response [34, 37, 38]. This remarkable regenerative capacity of the splenic niche may play a key role in initiating early hematopoiesis after allo-HSCT, even before the bone marrow has initiated the process. In other words, the spleen may act as a bridge until hematopoiesis is fully established in the bone marrow [39]. In this study, although neutrophil and platelet counts at day 50 showed no significant difference between the PBSCT and UCBT groups, ^18^F-FLT uptake in whole bone marrow tended to be lower in the UCBT group compared to the PBSCT group. This suggests that in the UCBT group, hematopoiesis in the spleen compensates for the lack of hematopoiesis in the systemic bone marrow after transplantation, and that recovery of the bone marrow niche may be more gradual in the UCBT group than in the PBSCT group. It is known that infusion of mesenchymal stem cells or endothelial progenitor cells induces recovery of the damaged bone marrow niche and promotes hematopoiesis [40–43]. The difference in time to bone marrow niche recovery between the PBSCT and UCBT groups may be due to differences in the type and amount of mesenchymal stem cells and endothelial progenitor cells contained in each group [44–47].

In addition, eosinophils and B lymphocytes increase during UCBT in the early post-engraftment phase [48–53]; in this study, they were elevated in the UCBT group compared with the PBSCT group beyond the 100-day milestone. Eosinophils may potentially attenuate allogeneic T-cell activation [54], while promoting B-cell survival, proliferation, and immunoglobulin secretion [55]. Moreover, cord blood is rich in interleukin-10-producing regulatory B cells, which suppresses T-cell immune responses and protect against the development of chronic GVHD [56]. During UCBT, the enhanced uptake of ^18^F-FLT in the spleen, a secondary lymphoid organ for B cells, after engraftment may be associated with the immune reconstitution that is unique to this transplantation modality.

This study has several limitations. First, the accuracy of extramedullary hematopoiesis within the hepatic domain was not investigated, as ^18^F-FLT is trapped in the liver because of glucuronidation [57, 58]. Second, we stratified post-transplant ^18^F-FLT uptake based on donor type; however, we failed to account for differences in conditioning regimen, primary disease, GVHD prophylaxis, or the presence of PIR/GVHD due to the small number of cases. Furthermore, the temporal progression of increased and decreased ^18^F-FLT uptake within the spleen during UCBT, before and after day 50, remains unclear. The relationship between peripheral blood chimerism and splenic hematopoiesis also remains undefined. These concerns require further investigation in an expanded patient cohort to determine the relationship between ^18^F-FLT uptake, various patient background determinants, and transplantation prognosis.

We have demonstrated that hematopoietic kinetics after allo-HSCT vary in the localization of hematopoiesis and its intensity when UCBT is contrasted with PBSCT. In particular, while hematopoiesis in the spleen before engraftment has been known, our study shows that the spleen plays an important role in maintaining systemic hematopoiesis after engraftment in UCBT. This may be due to the unique characteristics of the splenic niche. We believe that our findings provide valuable insights for the design of pre-transplant conditioning protocols that take into account the effects of the spleen and bone marrow on the niche, and for the development of novel therapies aimed at promoting niche recovery.

## Data Availability

The datasets generated during and/or analyzed during this study are available from the corresponding author on reasonable request.
